# Fine-scale genetic structure of the overwintering *Chilo suppressalis* in the typical bivoltine areas of northern China

**DOI:** 10.1371/journal.pone.0243999

**Published:** 2020-12-16

**Authors:** Ke-Xin Zhu, Shan Jiang, Lei Han, Ming-Ming Wang, Xing-Ya Wang

**Affiliations:** College of Plant Protection, Shenyang Agricultural University, Shenyang, Liaoning, China; CMAVE, USDA-ARS, UNITED STATES

## Abstract

The rice stem borer (RSB), *Chilo suppressalis* (Lepidoptera: Pyralidae), is an important agricultural pest that has caused serious economic losses in the major rice-producing areas of China. To effectively control this pest, we investigated the genetic diversity, genetic differentiation and genetic structure of 16 overwintering populations in the typical bivoltine areas of northern China based on 12 nuclear microsatellite loci. Moderate levels of genetic diversity and genetic differentiation among the studied populations were detected. Neighbour-joining dendrograms, Bayesian clustering and principal coordinate analysis (PCoA) consistently divided these populations into three genetic clades: western, eastern and northern/central. Isolation by distance (IBD) and spatial autocorrelation analyses demonstrated no correlation between genetic distance and geographic distance. Bottleneck analysis illustrated that RSB populations had not undergone severe bottleneck effects in these regions. Accordingly, our results provide new insights into the genetic relationships of overwintering RSB populations and thus contribute to developing effective management strategies for this pest.

## Introduction

The rice stem borer (RSB), *Chilo suppressalis* (Lepidoptera: Pyralidae), is a devastating rice pest in Asia, the Middle East, Europe and Oceania [1]. It attacks rice plants from the seedling stage to maturity and causes deadhearts and whiteheads [2,3]. In China, this pest is widely distributed in the main crop-producing areas and has caused severe economic losses in recent years. For example, RSB can damage seedlings to the point of withering and can cause losses of 526 kg per hectare in rice [4]. It has infested over 2.12 million hectares in northern China. Mainly, it occurs throughout the main rice-producing areas of Liaoning Province, Northeast China, which are among the typical bivoltine regions in China, and it can cause 5–20% rice yield loss [5,6].

RSB is a polyphagous pest with high fecundity and low dispersal capabilities [1,6]. There are differences in its reproductive generations among different regions. For example, one to four generations are produced in northern and southern Japan [1]. Similarly, in Northeast China, RSB has one generation annually in Heilongjiang and two generations in Liaoning, whereas four to five generations are produced in South China [7]. In general, high-instar RSB larvae overwinter in rice stems in Northeast China [8]. In mid-May, RSB adults begins to emerge and lays eggs in the upper bases and tips of rice leaves, gramineous crops and weeds. In mid-June, eggs hatch into the first generation of larvae and damage the rice. The second-generation larvae of RSB emerge in August and then begin to overwinter in rice stubble, rice straw and weeds in early October [9]. Currently, the use of chemical insecticides is still the primary control method for this species. However, RSB has developed resistance to some chemical insecticides such as triazophos, abamectin and deltamethrin [10]. Therefore, it is challenging to control this pest effectively.

Population genetics is a useful tool to illuminate the population genetic structure and to predict insect migration routes [11]. The genetic variation and genetic structure of species are being affected by climate change, human activities and migration behaviour [12–14]. Currently, many molecular markers are used to infer the biogeography and evolutionary patterns of species [15]. Due to their high codominance and polymorphism levels, microsatellite markers have been widely used in population genetics studies [16]. Previously, the genetic variation and population genetic structure of RSB have been investigated in China, Japan, South Korea and Iran based on random amplified polymorphic DNA (RAPD) [17], mitochondrial DNA and microsatellite markers [18–22]. Ishiguro and Tsuchida (2006) isolated and characterized four polymorphic microsatellite loci in RSB [18]. Although some molecular studies have been conducted on this destructive pest, it is still debated as to whether RBS populations have substantial population genetic differentiation and distinct genetic structure. For example, some previous studies showed that there was a high level of genetic differentiation and distinct phylogeographic structure in China based on RAPD, mitochondrial DNA genes (*COI*, *COII*, *16S*, *ND1* and *Cytb*) and microsatellite loci [17,19,21]. In contrast, moderate to high levels of genetic differentiation and no distinct population genetic structure were found in seven Chinese RBS populations based on microsatellite markers [20]. In addition, a low level of population genetic differentiation was detected in Northern Iran based on mtDNA *COI* gene sequences [22].

To our knowledge, RSB has relatively limited dispersal capacity, and adults disperse less than 1–3 km [23]. Discontinuous host distribution and the habitat fragmentation can increase the effects of random genetic drift and thus lead to local adaptations and population differentiation [24]. Due to the wide distribution range and low dispersal capacity of this pest, it is expected that genetic differentiation may occur among different local populations. To date, although several genetic studies have been performed on RSB, limited information is available on the genetic variability and fine-scale genetic structure of overwintering RBS in the typical bivoltine areas of China.

In this study, we investigated RSB population genetics in the typical bivoltine areas of northern China. Pure overwintering populations were collected in April 2019 through dissected the rice stubble. Twelve microsatellite loci were employed to investigate the genetic diversity, genetic differentiation and genetic structure of these RSB populations. The purpose of this study was to understand their population genetics and to provide a useful theoretical basis for developing effective control strategies for this pest.

## Materials and methods

### Sample collection and DNA extraction

**Fig 1:**
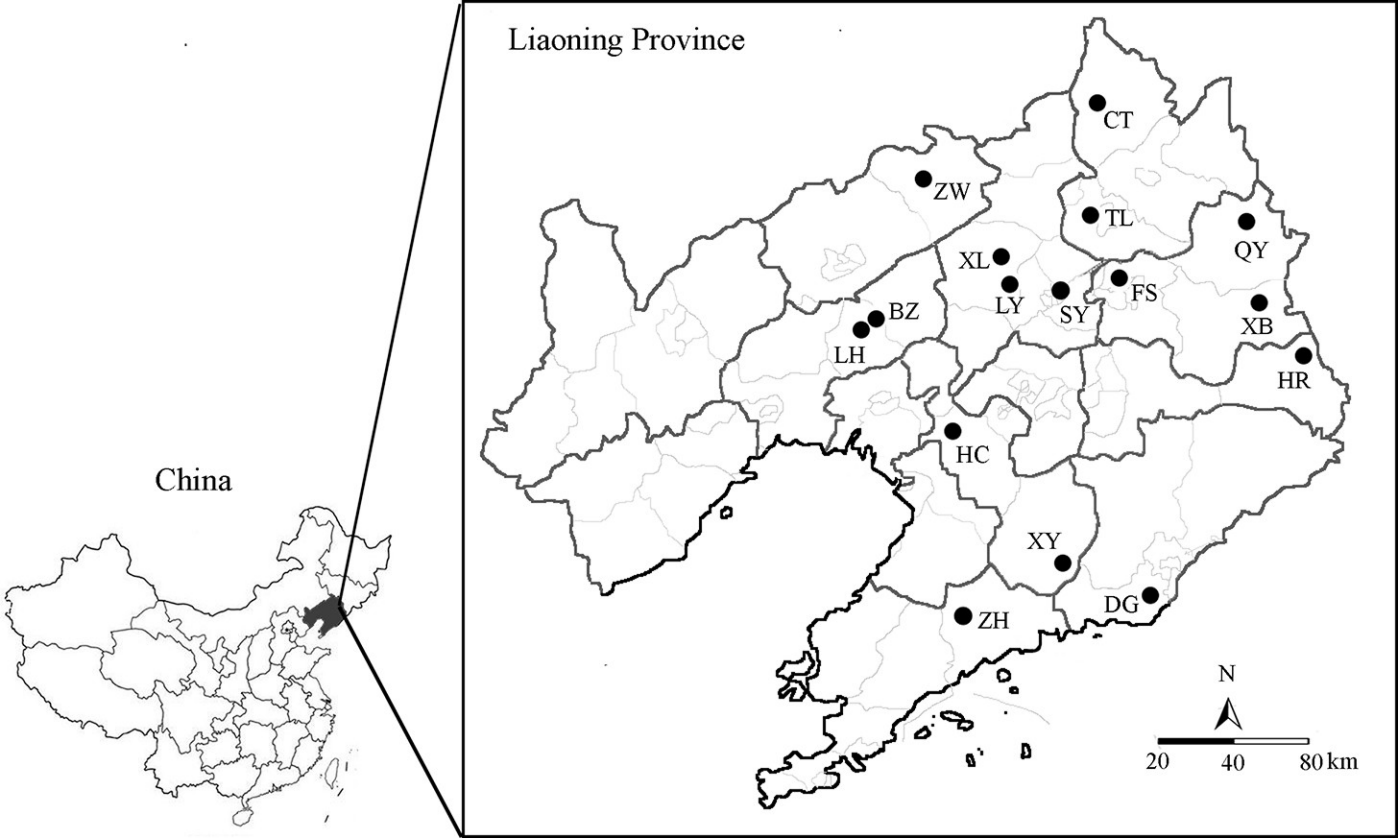
Sampling locations of 16 *Chilo suppressalis* populations in the typical bivoltine areas of northern China. See S1 Table for population abbreviations. The figure is modified from the graphic of Hu et al. (2015) [25].

By dissecting rice stems, a total of 367 RSB individuals were collected from 16 overwintering populations in Liaoning Province, Northeast China, from March to April 2019, and the numbers of individual samples per locality ranged from 8 to 30, averaging 22.6 (± 7.79) samples per locality (Fig 1, S1 Table). Each sampled overwintering individual of RSB was collected more than one meter away from the neighbouring individual to prevent overrepresentation of siblings. Samples were collected on private land with the landowners’ permission, and none of the field surveys involved protected or endangered species. All the RSB samples were stored in 95% ethanol at −20°C and deposited at the College of Plant Protection, Shenyang Agricultural University.

### DNA extraction, microsatellite amplification and genotyping

In this study, genomic DNA was extracted from individual samples using a Qiagen DNEasy Blood & Tissue Kit (Qiagen, Valencia, CA, USA). Twelve microsatellite loci developed specifically for RSB were used [18,26]. Among them, eleven loci (Cs248, Cs115, Cs218, Cs381, Cs117, Cs156, Cs62, Cs138, Cs133, Cs175 and Cs86) were chosen from Liu et al. [26], while the other locus (Cs11) was chosen from Ishiguro and Tsuchida [18]. These microsatellite loci were assigned unique fluorophores for DNA fluorescent labelling in PCRs. Each 20-μL PCR mixture consisted of 0.5 μl of genomic DNA, 1.0 unit of EasyTaq DNA polymerase, 2.5 mM dNTP mixture, 1×Easy Taq buffer, and 0.4 μM of each primer (labelled with either HEX or FAM). The PCR cycling conditions were as follows: 94°C for 4 min; 35 cycles at 94°C for 20 s, 52°C for 30 s, 72°C for 30 s; and a final extension at 72°C for 7 min. After amplification, an ABI 3730XL automatic sequencer (Applied Biosystems, Foster City, USA) was used to visualize the products at Sangon Biotech Co., Ltd. (Shanghai, China). Microsatellite allele analysis was conducted using GeneMapper 4.0 (Applied Biosystems).

### Data analyses

#### Genetic diversity

The probabilities of the presence of null alleles, large allele dropouts and scoring errors were detected using Micro-Checker 2.2.3 [27]. After Micro-Checker analysis, we estimated the global *F*_ST_ with and without using the excluding null alleles (ENA) correction conducted in the FreeNA programme [28]. Hardy-Weinberg equilibrium and genotypic linkage disequilibrium were calculated using Genepop 3.4 [29]. Correction for multiple tests was performed with Bonferroni corrections [30]. The mean number of alleles (*Na*), Shannon's information index (*I*), effective number of alleles (*Ne*), expected heterozygosity (*He*), unbiased expected heterozygosity (*uH*e) and observed heterozygosity (*H*_O_) were estimated using GenAlEx 6.5 software [31]. The inbreeding coefficient (*F*_IS_), fixation index (*F*_ST_) and allelic richness (*A*_*R*_) were calculated with FSTAT 2.9.3.2 [32]. Polymorphism information content (*PIC*) analysis was conducted by CERVUS 2.0 [33].

#### Genetic differentiation and gene flow

Population genetic differentiation was estimated using Wright’s *F*-statistic (*F*_ST_) in Arlequin 3.0, and the significance of the *F*_ST_ values was computed with 10,000 permutations [34]. The gene flow among populations (Nm), were obtained using the POPGENE (v 1.31) [35]. Heat maps were constructed in R statistical software 3.0.2 based on the *F*_ST_ values and geneflow (*N*_*m*_) values [36].

#### Population genetic structure

Five methods were used to infer the population genetic structure of RSB. First, an unrooted phylogenetic tree was constructed based on the neighbour-joining (NJ) method using POPTREE 2 [37]. Second, STRUCTURE 2.3 was used to identify genetic clusters based on the Bayesian approach [38]. Under the admixture and correlated allele frequencies among population models, we specified an initial range of potential genotype clusters (*K*) from one to sixteen [39,40]. The Markov chain Monte Carlo (MCMC) simulation was run ten times for each *K* for 500,000 iterations after a burn-in period of 50,000. The most likely number of clusters was determined based on the *Δ*K approach, which was implemented in Structure Harvester [38,41]. The final results were visualized as bar plots by finding the optimal alignment of the ten replicate analyses of the “best” *K* in CLUMPP 1.1 [42]. In addition, hierarchical STRUCTURE analyses were performed to identify any potential sub-structuring that may have been missed. Third, principal coordinate analysis (PCoA) based on the variance-covariance matrix calculated from the marker data was implemented in GenAlEx 6.5 [31]. Fourth, the hierarchical partitioning of genetic structure was tested using an analysis of molecular variance (AMOVA) in Arlequin 3.0 [34]. Fifth, to verify the hypothesis of isolation by distance (IBD), a Mantel test was used to test the correlation between pairwise *F*_ST_/(1-*F*_ST_) and ln (geographic distance) in GenAlEx 6.5 [31]. Finally, to study the relationship between geographic distance and genetic distance in more detail, we carried out a spatial autocorrelation analysis at 300 km based on IBD analysis following the approach of Smouse & Peakall [43] and Peakall et al. [44]. Moran's I spatial autocorrelation coefficients [45] were estimated from the sample locations and the microsatellite loci data using GenAlEx 6.5 [31].

#### Bottleneck effects, individual assignment and migrant detection

Population genetic bottlenecks were investigated using Bottleneck 1.2.02 [46] based on the tests of three alternative mutation models, the infinite allele model (IAM), the strict stepwise mutation model (SMM) and the two-phase mutation model (TPM). We also assessed the significance of the results using Wilcoxon’s test and used a qualitative descriptor of allele frequency distribution to discriminate between bottlenecked and stable populations. In addition, asymmetric pairwise measures of recent gene flows were estimated using partial Bayesian genetic approaches. Assignment tests and the detection of first-generation migrants for each population was implemented in GENECLASS 2.0 [47]. The assignment tests were performed using a partially Bayesian method [48]. Detection of first-generation migrants was detected by calculating the likelihood ratio L_home [49], using a calculation criterion based on allele frequencies as described by Paetkau et al. (1995) [50]. The probability value was calculated by 1,000 Monte Carlo simulations using the algorithm described by Paetkau et al. (2004) [49] with type I error rate of 0.01.

## Results

### Genetic diversity

In the present study, a total of 367 individuals from 16 populations in Liaoning Province, Northeast China, were genotyped using 12 microsatellite loci. Low null allele frequencies per locus were obtained, ranging from zero to 0.206 (S2 Table). No locus consistently displayed significant deviations from the Hardy–Weinberg expectations across the studied populations. Significant linkage disequilibrium was present in 55 of 367 tests (*a* = 0.05). In addition, the average *F*_ST_ values obtained with and without applying the ENA correction were 0.063 and 0.061, respectively, and these *F*_ST_ did not differ significantly (*F*_1,22_ = 0.014, *P* = 0.906). Thus, the presence of null alleles did not influence the *F*_ST_ estimation (S3 Table). The genetic variation among the 12 RSB microsatellite loci is summarized in S4 Table. Overall, the 12 microsatellite loci selected in this study were modestly polymorphic. The average number of alleles (*Na*) ranged from 2.750 in Cs117 to 17.500 in Cs381, with an average of 6.646. The effective number of alleles (*Ne*) ranged from 1.401–29.155 (average = 5.693). The Cs381 locus had the highest numbers of alleles and effective alleles. The observed heterozygosities ranged from 0.199 to 0.718, whereas the expected heterozygosities ranged from 0.287–0.967. The *PIC* values ranged from 0.888 in Cs248 to 0.380 in Cs133 and had a mean value of 0.616. The basic summary statistics of genetic variation among different RSB populations in the typical bivoltine areas of northern China are presented in S5 Table. The mean observed heterozygosity (*Ho* = 0.468) was similar to the mean expected heterozygosity (*He* = 0.595) in all populations. There were different levels of genetic variation among these populations. The unbiased expected heterozygosity (*uHe*) ranged from 0.543 in Beizhen (BZ) to 0.680 in Qingyuan (QY). The inbreeding coefficients (*F*_IS_) of the 12 microsatellite loci in all the RSB populations were very low (*F*_IS_ = 0.198), indicating an excess of heterozygotes, which reflected a balance in the sex ratio of these populations. The Liaoyang (LY) population had the highest level of genetic diversity, while the Fushun (FS) population had the lowest level of genetic diversity. The observed *Na* values ranged from 4.333 in FS to 8.500 in LY and had a mean value of 6.646. The *Ne* values across the microsatellite loci ranged from 2.959 in Xinglong (XL) to 4.414 in Liaoyang (LY).

### Genetic differentiation and gene flow

**Fig 2:**
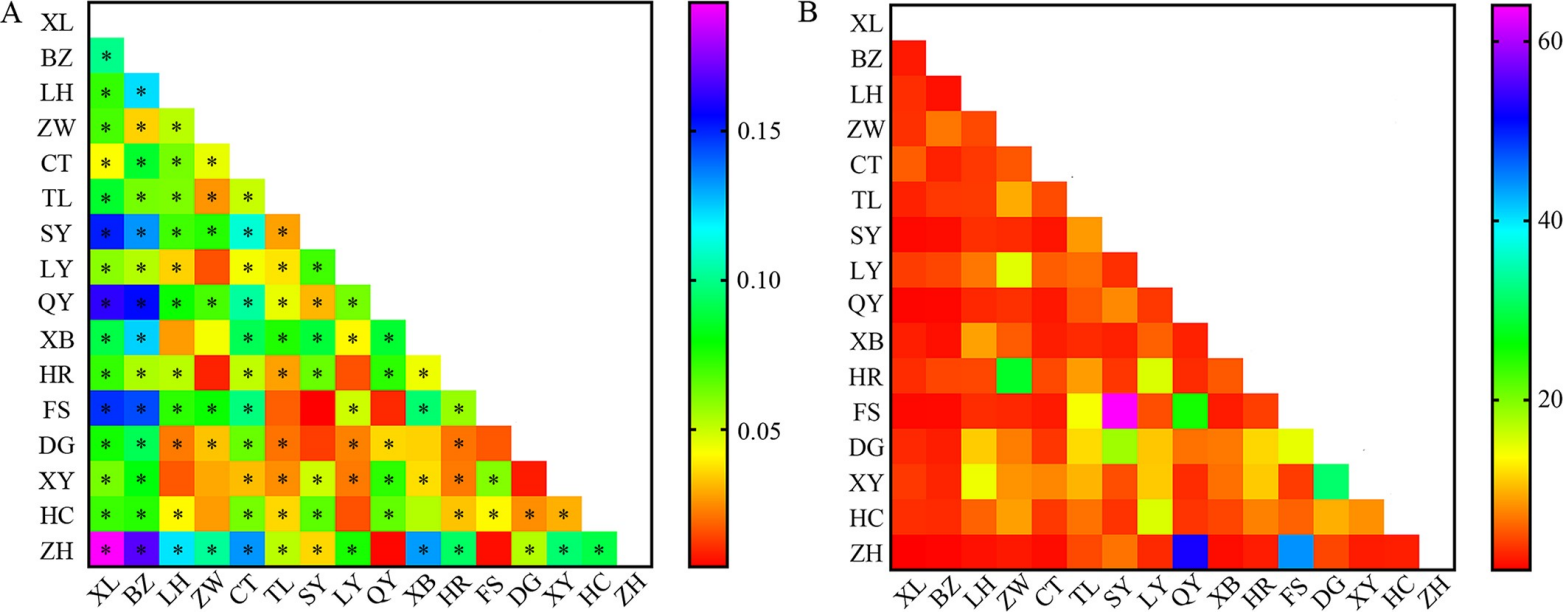
Heatmap of pairwise genetic differentiation (*F*_ST_) values and gene flow (*N*_*m*_) values between 16 populations of *Chilo suppressalis* in the typical bivoltine areas of northern China based on 12 microsatellite loci. *P < 0.05. The colours indicate *F*_ST_ or *N*_*m*_ values ranging from deep red (lower values) to purple (higher values). See S1 Table for population codes.

Overall, a moderate level of genetic differentiation among the populations was determined (global *F*_ST_ = 0.111). The pairwise *F*_ST_ values for genetic differentiation varied from 0.004 to 0.193 based on the nuclear data, and only 19 of the 136 pairwise comparisons were no significant genetic differentiation (S6 Table). Only a few pairwise *F*_ST_ comparisons did not show genetic differentiation on the basis of low pairwise *F*_ST_ values, such as those between Xiuyan (XY) and Zhangwu (ZW), LH (Linghai), and Donggang (DG) and those Liaoyang (LY) and Haicheng (HC), Zhangwu (ZW) and Huanren (HR). Overall, a low level of gene flow (global *N*_*m*_ = 2.002) was detected among the studied populations. Meanwhile, a high level of gene flow was found between some populations, such as Qingyuan (QY) and Zhuanghe (ZH) (Fig 2, S6 Table) (Fig 2, S6 Table).

### Population genetic structure

#### POPTREE analysis based on microsatellite data

**Fig 3:**
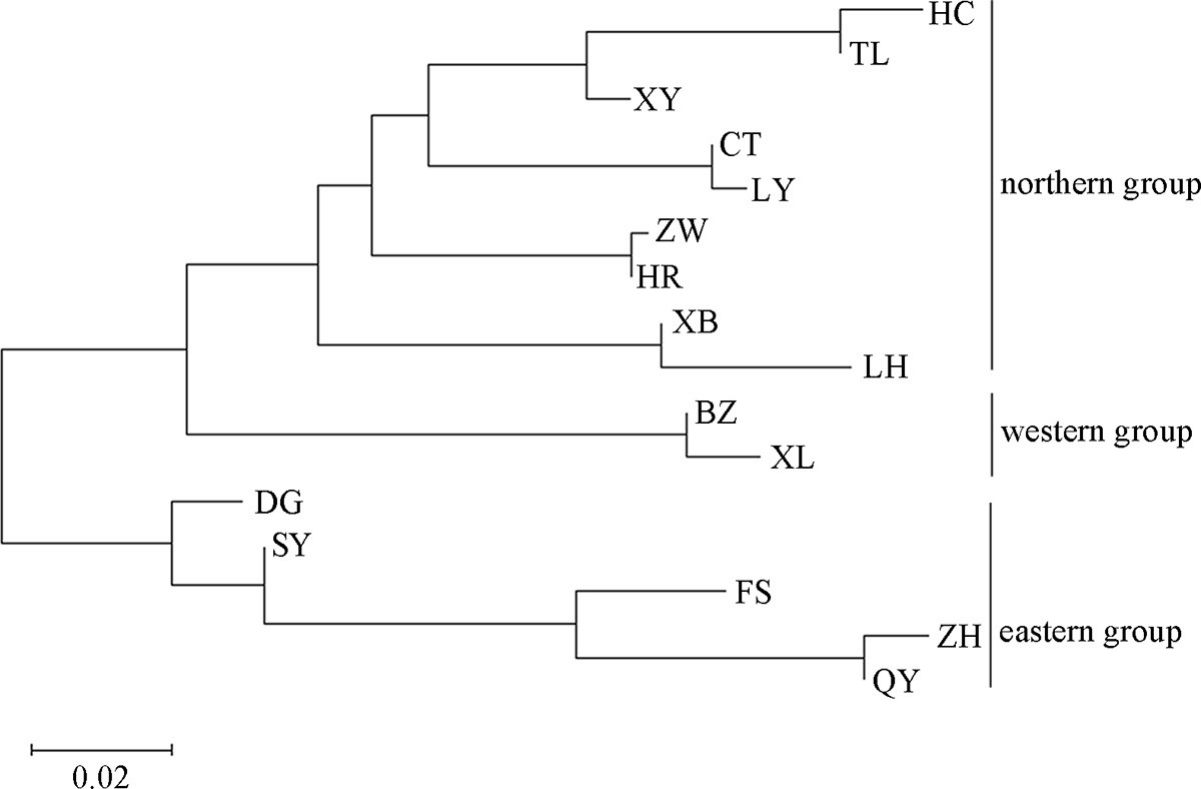
Neighbour-joining (NJ) phylogenetic tree based on 12 microsatellite datasets from 16 *Chilo suppressalis* populations in the typical bivoltine areas of northern China.

The unweighted NJ tree based on the microsatellite data of the 16 RSB populations defined three major groups (Fig 3). One group included five eastern populations, Shenyang (SY), Fushun (FS), Zhuanghe (ZH), Qingyuan (QY) and Donggang (DG). The second group included two western populations, Beizhen (BZ) and Xinglong (XL), and the third group included the remaining nine populations collected from the northern regions.

#### Bayesian clustering

**Fig 4:**
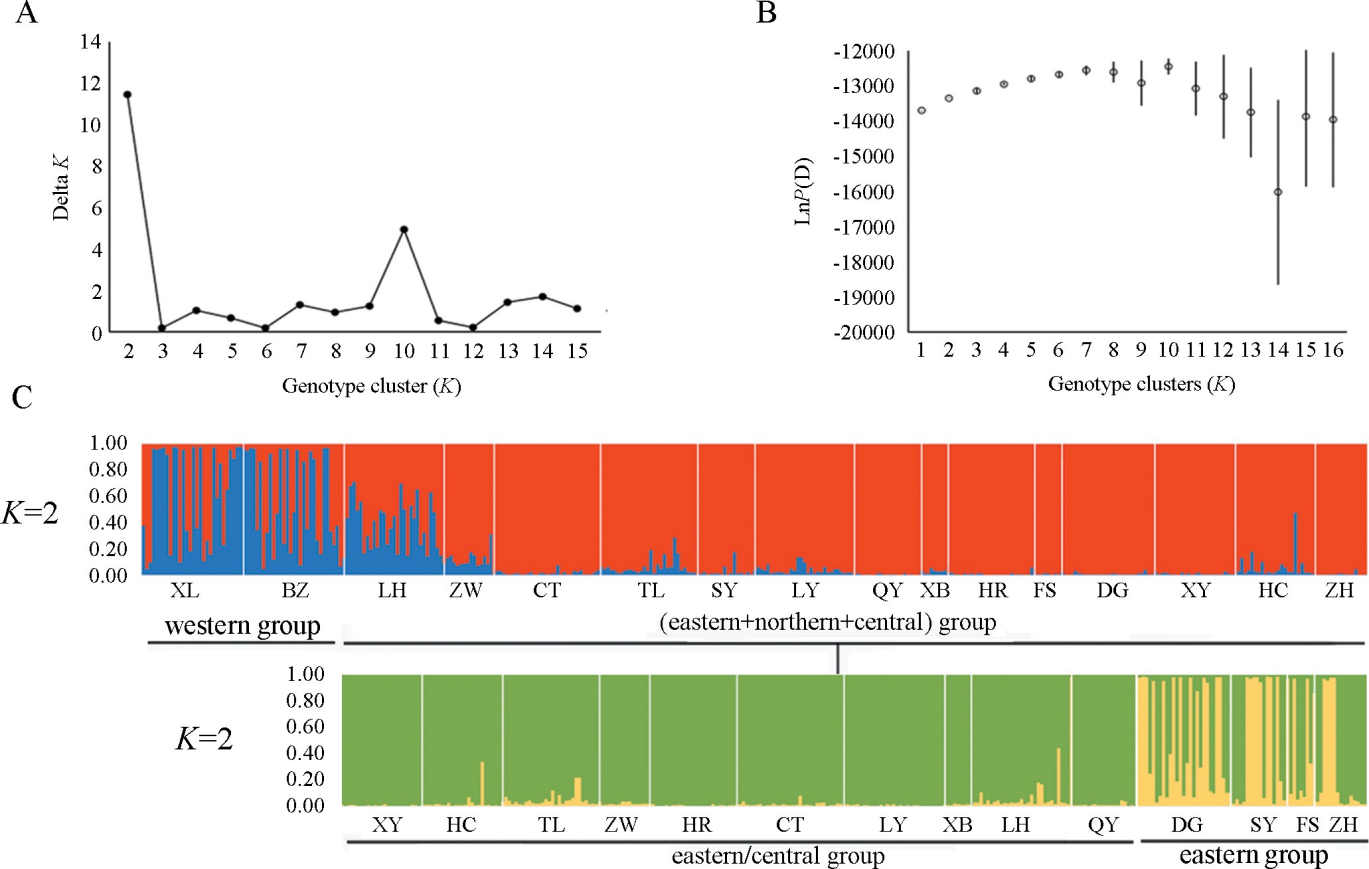
Hierarchical STRUCTURE analysis based on twelve microsatellite datasets from 16 *Chilo suppressalis* populations in the typical bivoltine areas of northern China. The initial STRUCTURE run split these populations into two main groups. *Δ*K (A) and ln *P (D)* (B) are plotted against the number of genetic clusters (*K*). The second genetic group was broken into two additional large groups (C). Each vertical coloured line represents the assignment proportion of membership in all the populations.

The Bayesian clustering model-based method allowed the degrees of genetic structure and admixture to be assessed among populations. An initial STRUCTURE analysis indicated a maximum Δ*K* at *K* = 2 (Fig 4), which could divide into two groups: one genetic group including the Xinglong (XL) and Beizhen (BZ) populations, which belong to the western region of Liaoning, and another group including the remaining 14 populations. We observed low Δ*K* values and choppiness of Δ*K* at higher *K* values (Fig 4A). In addition, there was no plateau whatsoever in the Ln P(D) values at *K* = 2 (Fig 4B). Then, a hierarchical STRUCTURE analysis for the second genetic group was conducted to detect further structure. The results indicated that a more fine-grained population structure was present at *K* = 2, and the subdivision still closely followed the geography. The Bayesian clustering method detected significant genetic clusters among the eastern populations, with Donggang (DG), Shenyang (SY), Fushun (FS) and Zhuanghe (ZH) in the second cluster and the remaining ten population grouped in the third group. This result was similar to that of the above NJ tree based on the same data.

#### Principal coordinate analysis (PCoA)

**Fig 5:**
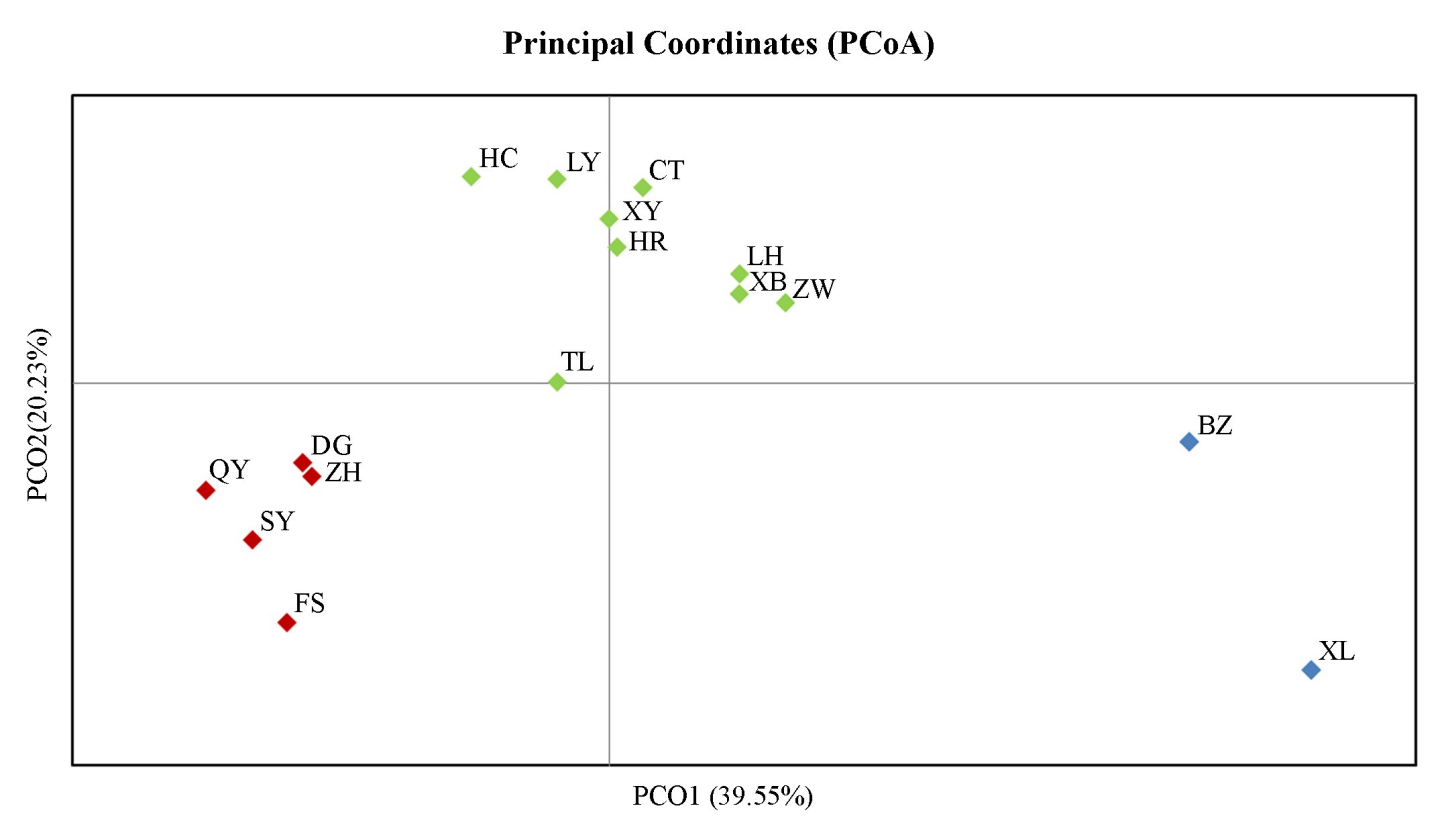
Principal coordinate analysis (PCoA) representing relationships among 16 *Chilo suppressalis* populations in the typical bivoltine areas of Northern China.

The population-based PCoA was performed based on Nei's genetic distance using allele frequencies of the twelve microsatellite loci in the 16 RSB populations (Fig 5). The PCoA results indicated a western–eastern–northern genetic structure, which was consistent with those shown above for the NJ tree (Fig 3) and Bayesian clustering (Fig 4). The first and second axes explained 39.55% and 20.23% of the overall variance, respectively. We also noticed that most geographically close populations resembled each other.

#### Analysis of molecular variance (AMOVA)

The results of the AMOVA for RSB based on twelve microsatellite loci in the typical bivoltine areas of northern China are shown in Table 1. First, the global AMOVA revealed that 11.09% of the total genetic variation could be explained by the variation among populations, and the remaining 88.91% could be explained by differences within populations. Second, the hierarchical AMOVA analysis demonstrated that 7.82% of the variation was explained by variation among regions, whereas the most variation (86.16%) was described by variation within populations.

**Table 1:** Hierarchical analysis of molecular variance (AMOVA) for *Chilo suppressalis* based on twelve microsatellite loci in the typical bivoltine areas of northern China.

Source of variation	*d*.*f*.	Sum of squares	Variance components	Percentage variation (%)	*F*-statistic
Global AMOVA					
Among populations	15	572.057	1.239 Va	11	*F*_ST_ = 0.111
Within populations	351	3486.815	9.934 Vb	89	
Total	366	4058.872	11.173	100	
Hierarchical AMOVA (*K* = 3)					
Among groups	2	36.531	0.061 Va	3.26	*F*_CT_ = 0.033***
Among populations within groups	13	66.668	0.076 Vb	4.02	*F*_SC_ = 0.042***
Within populations	718	1253.118	1.745 Vc	92.72	*F*_ST_ = 0.073***
Total	733	1356.317	1.882		

*d*.*f*., degrees of freedom

***P < 0.001: Significance level. Hierarchical AMOVA (*K* = 3): The first group includes two populations (XL and BZ) collected from western regions. The second group includes five populations (DG, SY, FS, ZH and QY) collected from eastern regions. The remaining 9 populations belong to the third group.

#### Correlation between geographic distance and genetic distance

**Fig 6:**
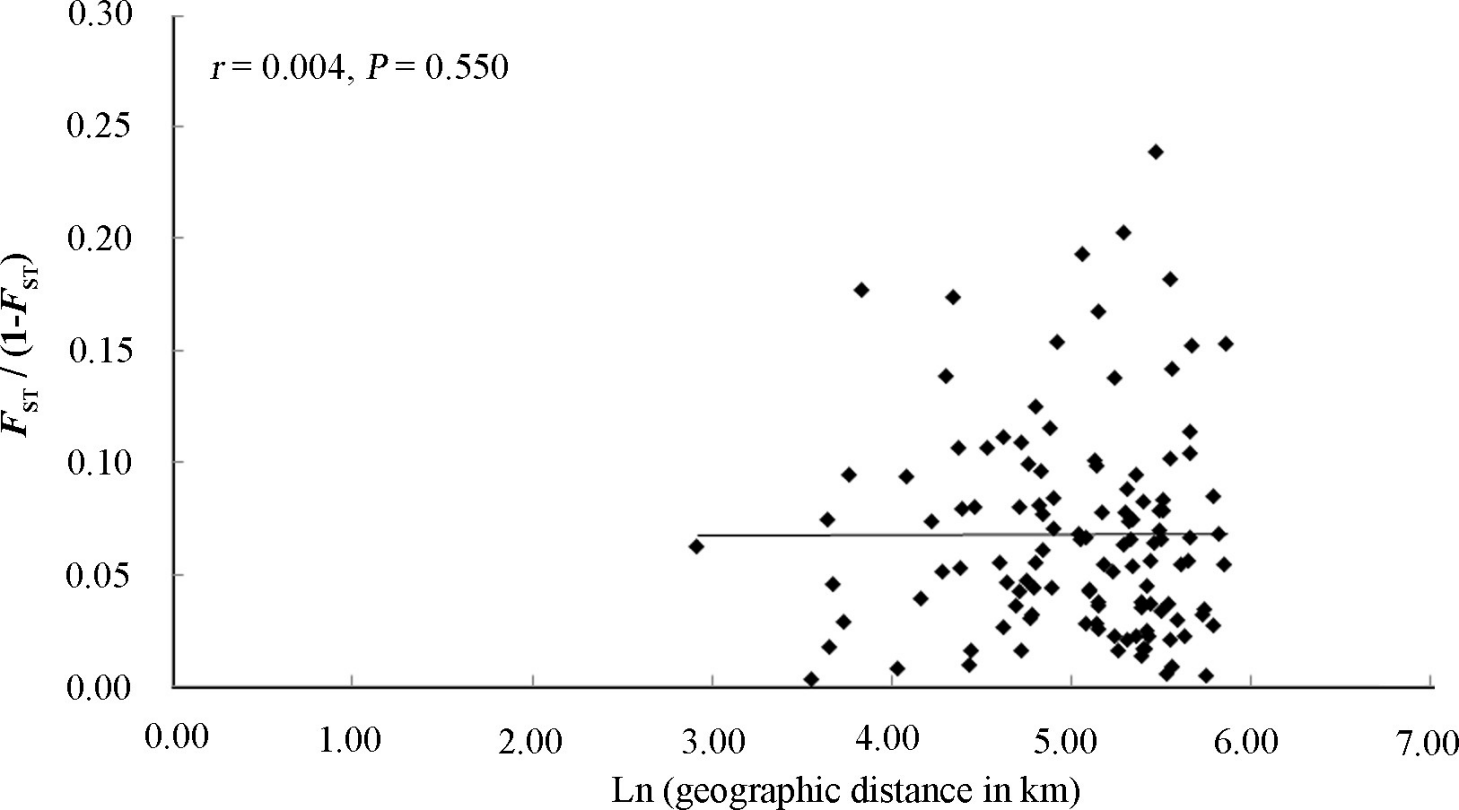
Correlation analysis between pairwise *F*_ST_/1-*F*_ST_ values and ln (geographic distance (km)) in the typical bivoltine areas of northern China based on twelve microsatellite loci.

**Fig 7:**
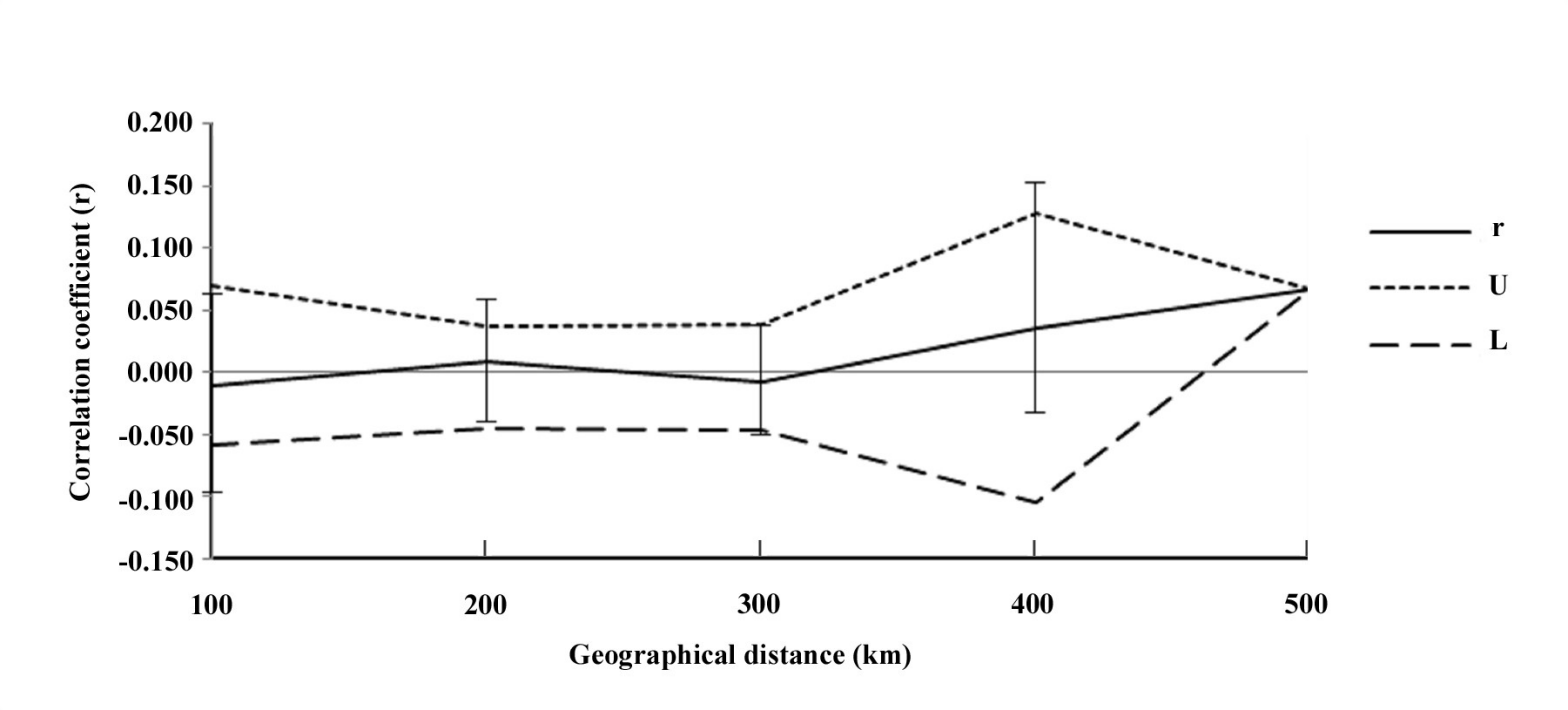
Correlograms of spatial autocorrelation for genetic populations of *Chilo suppressalis* in the typical bivoltine regions in northern China. *r* represents genetic correlation values, which are plotted as a function of distance. Upper (U) and low (L) are the bounds of a 95% CI for r generated under the null hypotheses of random geographic distribution. Autocorrelation for distance was performed with class sizes of 300 km.

A Mantel test for IBD was conducted to estimate the correlation between the genetic distance and geographic distance in RSB. If the dispersal of RSB is limited by distance, a positive correlation between geographic distance and genetic distance would be expected. In this study, no significant positive relationships between genetic and geographic distances were found (*r* = 0.004, *P* = 0.550, Fig 6). Therefore, the RSB populations in the typical bivoltine areas of northern China showed no pattern of IBD. The results of the spatial autocorrelation analysis showed that geographic distance and genetic distance did not correlate (*P* > 0.05, 999 randomizations) (Fig 7). There was no rejection of the null hypothesis of random distribution on most scales.

#### Bottleneck effect analysis

The results of bottleneck analysis based on twelve microsatellite loci in the typical bivoltine areas of northern China are summarized in Table 2. Under the IAM, TPM and strict SMM, there was no significant heterozygote excess in any of the populations. Moreover, the results were consistent with a normal L-shaped distribution of allelic frequency, indicating that RSB populations had not experienced a severe bottleneck in these regions.

**Table 2:** Wilcoxon signed-rank test for mutation–drift equilibrium estimated for *Chilo suppressalis* based on twelve microsatellite loci in the typical bivoltine areas of northern China.

Population	Heterozygosity excess *P*-value			
	IAM	TPM	SMM	Mode shift
XY	0.765	0.983	0.100	L
HC	0.285	0.912	0.995	L
TL	0.661	0.954	0.998	L
ZW	0.190	0.661	0.954	L
HR	0.689	0.961	0.999	L
CT	0.055	0.788	0.997	L
LY	0.604	0.935	0.996	L
XB	0.087	0.382	0.793	S
LH	0.212	0.455	0.945	L
BZ	0.485	0.935	0.996	L
XL	0.102	0.485	0.945	L
DG	0.741	0.974	0.999	L
SY	0.151	0.689	0.954	L
FS	0.088	0.285	0.715	L
ZH	0.235	0.485	0.788	L
QY	0.055	0.455	0.898	L

*P* is the test result for heterozygosity excess, *P < 0.05, **P < 0.01; IAM: Infinite allele model; TPM: Two-phase model; SMM: Stepwise mutation model; L: Normal L-shaped distribution; S: Shifted mode.

#### Individual assignment and migrant detection

The results of GENECLASS analysis revealed that the assignment rate of individuals to their original location was 89.10% (40/367). The remaining 40 individuals (10.90%) were putative migrants and were evenly distributed in the typical bivoltine areas of northern China. In addition, a total of 13 potentially first-generation migrants were detected in the studied populations, and only four first-generation migrants were found in the Donggang (DG) population, which indicated that the low level of gene flow and non-directional spread occurred among most RSB populations in the typical bivoltine areas of northern China (Table 3).

**Table 3:** Results of the assignment test and detection of first-generation migrants among *Chilo suppressalis* individuals, with source populations listed by column and recipient populations by row.

Pop.	XL	BZ	LH	ZW	CT	TL	SY	LY	QY	XB	HR	FS	DG	XY	HC	ZH
XL	27		1							1			2			
BZ		24	(1)	2(1)			1	(1)	1	1			1			
LH	2		27		1								(1)			
ZW				14		(1)	(1)								1	
CT					29	1				1(1)			1			
TL		1	1			24	1			1	1(1)					(1)
SY		1					14	1	1	(1)		(1)				
LY			(1)		1(1)			25		1(1)	2				1	
QY									19							1(1)
XB										8			(1)			
HR				1							23		1			
FS												8				
DG							3			1		1(1)	21	1	1	
XY											2		1(1)	21		
HC		1						(1)	1				1(1)		18	3
ZH																16

## Discussion

### Moderate levels of genetic diversity and genetic differentiation

The study of intraspecific variation can not only uncover new putative species but also allow inference of evolutionary origin and population history [51]. In this study, a moderate level of genetic diversity of RSB in the typical bivoltine areas of northern China (*He =* 0.648) was found. This is in agreement with the results of Meng et al. [19] and Liu et al. [20]. The values of *He* in these 18 Chinese populations ranged from 0.591 to 0.725 [19], and the values of *He* in South China ranged from 0.625 to 0.833 [20]. Moreover, the *F*_IS_ values of four RSB populations, Tieling (TL), Linghai (LH), Xinglong (XL) and Zhuanghe (ZH), were slightly less than zero, indicating an excess of heterozygotes (S5 Table). Notably, it is worth noting that the Qingyuan (QY) population had the highest level of genetic diversity (*Ho* = 0.602, *He* = 0.662). A large number of RSB could migrate from southern to northern China annually, and this genetic exchange could lead to high heterozygosity in the RSB populations, enhancing the adaptability of these populations towards changing environmental conditions and thus leading to the high level of genetic diversity observed in the populations in these areas.

We found a moderate level of genetic differentiation among these overwintering RSB populations in the typical bivoltine areas of northern China based on microsatellite data, which was consistent with previously reported findings. For example, another group found a moderate to high level of genetic differentiation and no genetic structure in seven Chinese RSB populations by using microsatellite markers (Liu et al., 2013) [20]. Tang et al. (2016) investigated the genetic diversity among 44 populations of RSB in China with the mtDNA *Cytb* gene and found significant genetic differentiation among the populations [21]. The limited dispersal capacity of RSB might increase the effects of random genetic drift, leading to local adaptation and population differentiation at large geographic scales. In contrast, Shayanmehr and Yoosefi-Lafooraki (2016) found a low level of genetic diversity and a high level of gene flow between populations in Northern Iran based on mtDNA *COI* gene sequences [22]. The differences in population differentiation obtained with these two different genetic markers could be mainly attributed to the different mutation rates of microsatellite and mtDNA markers [20]. Additionally, it is generally assumed that the *Nm* values indicate less than 2 migrants per generation, which suggests that the observed differentiation levels were the result of migration–drift equilibrium. If *N*_*m*_ > 4, then the local populations belong to one panmictic population [19]. In our study, the low values of gene flow (*N*_*m*_ = 2.002) were found among most of the populations, which attributed to the habitat barriers or environmental isolation in these regions [52].

### Distinct nuclear population structure

Understanding the genetic structure of species helps to provide insights into the evolutionary and ecological processes [53]. In this study, we revealed a distinct RSB population genetic structure in the typical bivoltine areas of northern China based on neighbour-joining dendrograms, STRUCTURE analysis and principal coordinates analysis (PCoA). The populations can be divided into three genetic groups: western, eastern and northern/central clade. The first group included two western populations, Beizhen (BZ) and Xinglong (XL). The second group included four eastern populations, Shenyang (SY), Fushun (FS), Zhuanghe (ZH) and Donggang (DG). The remaining ten populations were clustered into the third group. To the best of our knowledge, the main rice-producing areas of Liaoning include the Yellow Sea plain areas along the southeast, eastern mountainous areas, Liaohe plain areas and western mountainous rice growing areas. The eastern mountainous areas of Liaoning belong to Changbaishan Mountain, and the western mountains of Liaoning are the transition belt of the Mongolia Plateau to Liaohe plain. Therefore, the habitat barriers of the eastern and western mountains have restricted gene flow among the RSB populations, causing environmental isolation and population genetic differentiation. In contrast, due to the lack of habitat fragmentation and the similarity of the climate and soil composition, the RSB populations in the middle and northern parts of Liaoning clustered together. These results were similar to those of Meng et al. [18], which indicated that RSB has substantial genetic heterogeneity across large geographic distances, and the populations can be divided into three clades in China: a central China clade, a northern/northeastern China clade and a southwestern China clade [18]. In general, IBD is strong in low-mobility species but not pronounced in moderately mobile species [54]. Here, both IBD and the fine-scale spatial autocorrelation analyses demonstrated no correlation between geographical distance and genetic differentiation. Although RSB has limited active dispersal ability, this moth pest can be carried long distances by wind or human transportation. Therefore, these lines of evidence support limited gene flow, moderate level of genetic differentiation and distinct phylogeographic structure of RSB populations in the typical bivoltine areas of northern China.

### Pest management implications

RSB is one of the most destructive rice pests in China. In the past few decades, chemical control has remained the most efficient primary means of controlling this pest. Unfortunately, it has developed resistance to some kinds of insecticides, and insecticide resistance loci may show selection footprints [10]. Therefore, a survey of the resistance to insecticides and the distribution of resistance genes in RSB at a large geographic scale in China will contribute to determining the level of insecticide resistance and will guide the use of insecticides. Furthermore, understanding the genetic structure, dispersal ability, migration route and population demography of insect species is vital to population biology and evolutionary study and to the effective implementation of monitoring forecast systems [55]. Therefore, we will investigate the genetic variation, geneflow and genetic structure of RSB at the whole-genome level, aiming to uncover its genetic evolutionary and demographic history and thus to understand the mechanisms underlying its adaptation to the environment. These projects will be implemented as soon as possible so that effective management strategies can be developed for this pest.

## Conclusions

Our microsatellite results illustrated a moderate level of genetic diversity and a moderate level of genetic differentiation among the RSB populations in the typical bivoltine areas of northern China. We also noted a distinct population genetic structure, which divided these populations into three clades: western, eastern and northern/centra. IBD and spatial autocorrelation analyses demonstrated no correlation between genetic and geographic distance, while bottleneck effect analysis indicated that RSB had not undergone a severe bottleneck in these regions. The results of GENECLASS analysis led us to speculate that low level of gene flow and non-directional spread occurred among most RSB populations located in the typical bivoltine areas of northern China. Our results provide new insights into the fine-scale genetic structure of overwintering RSB in the typical bivoltine areas of northern China and contribute to understanding the dispersal pattern of this species in China.

## Supporting information

S1 Table Sampling information of *Chilo suppressalis* in the typical bivoltine areas of Northern China. (DOC)

S2 Table Null allele frequency for each locus of *Chilo suppressalis*. (DOC)

S3 Table Estimates of *F*_ST_ without and with the excluding null alleles (ENA) correction for each locus of *Chilo suppressalis*. (DOC)

S4 Table Genetic variations in twelve microsatellite loci of *Chilo suppressalis* in the typical bivoltine areas of Northern China. (DOC)

S5 Table Population genetic diversity of *Chilo suppressalis* based on twelve microsatellite loci in the typical bivoltine areas of northern China. (DOC)

S6 Table Pairwise *F*_ST_ (below the diagonal) and gene flow (*Nm*) values (above the diagonal) values between sixteen populations of *Chilo suppressalis* in the typical bivoltine areas of northern China based on twelve microsatellite loci. (DOC)

S1 Data (XLSX)
