# Supplementary material for: Fine-scale genetic structure of the overwintering Chilo suppressalis in the typical bivoltine areas of northern China
Source: PLoS One. 2020 Dec 16;15(12):e0243999. doi: 10.1371/journal.pone.0243999 (PMC7743936; doi:10.1371/journal.pone.0243999)
Supplement: S1 Table — (DOC) [file pone.0243999.s001.doc]

**S1 Table. Sampling information of *Chilo suppressalis* in the typical bivoltine areas of Northern China**

| Sampling locations | Code | SSR | Insect stage | Latitude / Longitude | Collection date | Altitude (m) |
| --- | --- | --- | --- | --- | --- | --- |
| Xinglong, Xinmin | XL | 30 | Larva | 41.973°N 123.044°E | October 2018 | 30 |
| Beizhen, Jinzhou | BZ | 30 | Larva | 41.543°N 121.949°E | April 2019 | 65 |
| Linghai, Jinzhou | LH | 25 | Larva | 41.517°N 121.864°E | October 2018 | 15 |
| Zhangwu, Fuxin | ZW | 15 | Larva | 42.424°N 122.544°E | April 2019 | 110 |
| Changtu, Tieling | CT | 32 | Larva | 42.915°N 123.782°E | April 2019 | 90 |
| Tieling, Tieling | TL | 29 | Larva | 42.191°N 123.703°E | April 2019 | 61 |
| Shenhe, Shenyang | SY | 17 | Larva | 41.823°N 123.573°E | March 2019 | 50 |
| Taizihe, Liaoyang | LY | 30 | Larva | 41.813°N 123.106°E | April 2019 | 18 |
| Qingyuan, Fushun | QY | 20 | Larva | 42.108°N 124.967°E | April 2019 | 201 |
| Xinbin, Fushun | XB | 8 | Larva | 41.721°N 125.080°E | April 2019 | 329 |
| Huanren, Benxi | HR | 26 | Larva | 41.448°N 125.382°E | April 2019 | 450 |
| Shuncheng, Fushun | FS | 8 | Larva | 41.909°N 123.979°E | April 2019 | 134 |
| Donggang, Dandong | DG | 28 | Larva | 39.889°N 124.106°E | April 2019 | 4 |
| Xiuyan, Anshan | XY | 24 | Larva | 40.141°N 123.525°E | April 2019 | 60 |
| Haicheng, Anshan | HC | 24 | Larva | 40.894°N 122.529°E | April 2019 | 13 |
| Zhuanghe, Dalian | ZH | 16 | Larva | 39.842°N 122.678°E | April 2019 | 70 |
