# Supplementary material for: Fine-scale genetic structure of the overwintering Chilo suppressalis in the typical bivoltine areas of northern China
Source: PLoS One. 2020 Dec 16;15(12):e0243999. doi: 10.1371/journal.pone.0243999 (PMC7743936; doi:10.1371/journal.pone.0243999)
Supplement: S2 Table — (DOC) [file pone.0243999.s002.doc]

**S2 Table. Null allele frequency for each locus of *Chilo suppressalis***

| Locus | Null allele frequency |
| --- | --- |
| Cs248 | 0.162 |
| Cs175 | 0.149 |
| Cs218 | 0.024 |
| Cs381 | 0.119 |
| Cs86 | 0.117 |
| Cs133 | 0.098 |
| Cs138 | 0.206 |
| Cs62 | 0.160 |
| Cs156 | 0.022 |
| Cs115 | 0.045 |
| Cs117 | 0.000 |
| Cs11 | 0.145 |
