# Supplementary material for: Fine-scale genetic structure of the overwintering Chilo suppressalis in the typical bivoltine areas of northern China
Source: PLoS One. 2020 Dec 16;15(12):e0243999. doi: 10.1371/journal.pone.0243999 (PMC7743936; doi:10.1371/journal.pone.0243999)
Supplement: S3 Table — (DOC) [file pone.0243999.s003.doc]

***S3 Table. Estimates of F_ST_ without and with the excluding null alleles (ENA) correction for each locus of*** Chilo suppressalis

| Locus | *F*_ST_ not using ENA | *F*_ST_ using ENA |
| --- | --- | --- |
| Cs248 | 0.035 | 0.032 |
| Cs175 | 0.091 | 0.086 |
| Cs218 | 0.024 | 0.023 |
| Cs381 | 0.035 | 0.029 |
| Cs86 | 0.157 | 0.141 |
| Cs133 | 0.044 | 0.060 |
| Cs138 | 0.057 | 0.055 |
| Cs62 | 0.051 | 0.042 |
| Cs156 | 0.035 | 0.038 |
| Cs115 | 0.065 | 0.064 |
| Cs117 | 0.101 | 0.101 |
| Cs11 | 0.057 | 0.060 |

The excluding null alleles (ENA).
