# Supplementary material for: Fine-scale genetic structure of the overwintering Chilo suppressalis in the typical bivoltine areas of northern China
Source: PLoS One. 2020 Dec 16;15(12):e0243999. doi: 10.1371/journal.pone.0243999 (PMC7743936; doi:10.1371/journal.pone.0243999)
Supplement: S4 Table — (DOC) [file pone.0243999.s004.doc]

**S4 Table. Genetic variation information of twelve microsatellite loci of *Chilo suppressalis* in the typical bivoltine areas of Northern China**

| Loci | *Na* | *Ne* | *I* | *Ho* | *He* | *uHe* | *F*_ST_ | *F*_IT_ | *F*_IS_ | *Nm* | *Hs* | *A_R_* | *r* | *PIC* |
| --- | --- | --- | --- | --- | --- | --- | --- | --- | --- | --- | --- | --- | --- | --- |
| Cs248 | 10.438 | 6.432 | 2.043 | 0.551 | 0.840 | 0.864 | 0.061 | 0.384 | 0.344 | 3.877 | 0.873 | 5.981 | 0.162 | 0.888 |
| Cs175 | 3.250 | 2.053 | 0.825 | 0.330 | 0.488 | 0.502 | 0.114 | 0.402 | 0.325 | 1.941 | 0.506 | 2.546 | 0.149 | 0.452 |
| Cs218 | 6.000 | 2.524 | 1.195 | 0.611 | 0.584 | 0.604 | 0.053 | 0.009 | -0.046 | 4.501 | 0.603 | 3.655 | 0.024 | 0.573 |
| Cs381 | 17.500 | 11.680 | 2.597 | 0.687 | 0.904 | 0.929 | 0.063 | 0.288 | 0.241 | 3.725 | 0.936 | 7.546 | 0.119 | 0.965 |
| Cs86 | 4.250 | 2.631 | 1.079 | 0.387 | 0.577 | 0.596 | 0.177 | 0.448 | 0.330 | 1.166 | 0.603 | 3.239 | 0.117 | 0.629 |
| Cs133 | 3.813 | 1.686 | 0.722 | 0.300 | 0.382 | 0.395 | 0.078 | 0.275 | 0.213 | 2.937 | 0.397 | 2.533 | 0.098 | 0.380 |
| Cs138 | 3.563 | 2.041 | 0.827 | 0.196 | 0.482 | 0.495 | 0.095 | 0.631 | 0.593 | 2.387 | 0.504 | 2.539 | 0.206 | 0.459 |
| Cs62 | 5.563 | 2.303 | 1.074 | 0.299 | 0.524 | 0.541 | 0.094 | 0.484 | 0.430 | 2.411 | 0.550 | 3.353 | 0.160 | 0.579 |
| Cs156 | 8.063 | 2.879 | 1.357 | 0.636 | 0.620 | 0.637 | 0.051 | 0.025 | -0.027 | 4.673 | 0.637 | 3.973 | 0.022 | 0.624 |
| Cs115 | 9.250 | 4.890 | 1.765 | 0.681 | 0.759 | 0.779 | 0.089 | 0.182 | 0.102 | 2.546 | 0.782 | 5.114 | 0.045 | 0.835 |
| Cs117 | 2.750 | 1.422 | 0.461 | 0.337 | 0.259 | 0.266 | 0.128 | -0.138 | -0.305 | 1.710 | 0.264 | 1.895 | 0.000 | 0.264 |
| Cs11 | 5.313 | 3.675 | 1.428 | 0.595 | 0.720 | 0.740 | 0.077 | 0.236 | 0.173 | 3.007 | 0.744 | 4.124 | 0.145 | 0.748 |
| Mean | 6.646 | 3.685 | 1.281 | 0.468 | 0.595 | 0.612 | 0.090 | 0.269 | 0.198 | 2.907 | 0.617 | 5.981 | 0.104 | 0.616 |

Abbreviations: *Na*, Observed number of alleles; *Ne*, Effective number of alleles; *I*, Shannon's information index; *Ho*, Observed heterozygosity; *He*, Expected heterozygosity; *uHe*, Unbiased expected heterozygosity; *F*_ST_, Fixation index; *F*_IS_, Inbreeding coefficient; *H*_S_, gene diversity; *A_R_*, Allelic Richness; *r*, frequency of null alleles. *PIC*, Polymorphism Information Content.
