# Supplementary material for: Fine-scale genetic structure of the overwintering Chilo suppressalis in the typical bivoltine areas of northern China
Source: PLoS One. 2020 Dec 16;15(12):e0243999. doi: 10.1371/journal.pone.0243999 (PMC7743936; doi:10.1371/journal.pone.0243999)
Supplement: S5 Table — (DOC) [file pone.0243999.s005.doc]

**S5 Table. Population genetic diversity of *Chilo suppressalis* based on twelve microsatellite loci in the typical bivoltine areas of northern China**

| Pop. | *Na* | *Ne* | *I* | *Ho* | *He* | *uHe* | *F*_ST_ | *F*_IS_ | *H*_S_ | *A*_P_ |
| --- | --- | --- | --- | --- | --- | --- | --- | --- | --- | --- |
| XY | 8.167 | 4.265 | 1.368 | 0.480 | 0.598 | 0.612 | 0.061 | 0.344 | 0.615 | 0.667 |
| HC | 6.500 | 3.552 | 1.244 | 0.433 | 0.574 | 0.586 | 0.114 | 0.325 | 0.589 | 0.500 |
| TL | 8.083 | 3.831 | 1.411 | 0.429 | 0.636 | 0.648 | 0.053 | -0.046 | 0.652 | 0.750 |
| ZW | 6.000 | 3.717 | 1.281 | 0.390 | 0.598 | 0.621 | 0.063 | 0.241 | 0.631 | 0.333 |
| HR | 7.667 | 3.701 | 1.333 | 0.463 | 0.598 | 0.610 | 0.177 | 0.330 | 0.613 | 0.583 |
| CT | 7.500 | 3.938 | 1.367 | 0.505 | 0.611 | 0.621 | 0.078 | 0.213 | 0.623 | 0.250 |
| LY | 8.500 | 4.414 | 1.411 | 0.500 | 0.611 | 0.622 | 0.095 | 0.593 | 0.624 | 0.583 |
| XB | 4.417 | 3.098 | 1.118 | 0.415 | 0.560 | 0.598 | 0.094 | 0.430 | 0.612 | 0.000 |
| LH | 6.833 | 3.785 | 1.303 | 0.482 | 0.586 | 0.596 | 0.051 | -0.027 | 0.598 | 0.333 |
| BZ | 6.250 | 2.980 | 1.123 | 0.423 | 0.532 | 0.543 | 0.089 | 0.102 | 0.545 | 0.167 |
| XL | 5.167 | 2.959 | 1.099 | 0.402 | 0.532 | 0.546 | 0.128 | -0.305 | 0.548 | 0.083 |
| DG | 8.167 | 4.009 | 1.346 | 0.473 | 0.594 | 0.606 | 0.077 | 0.173 | 0.609 | 0.333 |
| SY | 6.583 | 3.976 | 1.352 | 0.480 | 0.628 | 0.648 | 0.061 | 0.344 | 0.653 | 0.167 |
| FS | 4.333 | 2.968 | 1.146 | 0.492 | 0.609 | 0.654 | 0.114 | 0.325 | 0.667 | 0.000 |
| ZH | 5.667 | 3.761 | 1.212 | 0.512 | 0.589 | 0.609 | 0.053 | -0.046 | 0.613 | 0.083 |
| QY | 6.500 | 3.999 | 1.384 | 0.602 | 0.662 | 0.680 | 0.063 | 0.241 | 0.683 | 0.167 |
| Mean | 6.646 | 3.685 | 1.281 | 0.468 | 0.595 | 0.612 | 0.090 | 0.198 | 0.615 | 0.312 |

Abbreviations: *N*_a_, observed number of alleles; *N*_e_, effective number of alleles; *I*, Shannon’s information index, *H*_o_, observed heterozygosity, *H*_e_, expected heterozygosity; uH_E_, unbiased expected heterozygosity; *F*, fixation index; *F*_IS_, inbreeding index; *H*_S_, gene diversity; *A*_P_, number of private alleles.
