# Supplementary material for: Fine-scale genetic structure of the overwintering Chilo suppressalis in the typical bivoltine areas of northern China
Source: PLoS One. 2020 Dec 16;15(12):e0243999. doi: 10.1371/journal.pone.0243999 (PMC7743936; doi:10.1371/journal.pone.0243999)
Supplement: S6 Table — (DOC) [file pone.0243999.s006.doc]

**S6 Table. Pairwise *F*_ST_ (below diagonal) and gene flow (*Nm*) values (above diagonal) among sixteen populations of *Chilo suppressalis* in the typical bivoltine areas of northern China based on twelve microsatellite loci**

|  | XL | BZ | LH | ZW | CT | TL | SY | LY | QY | XB | HR | FS | DG | XY | HC | ZH |
| --- | --- | --- | --- | --- | --- | --- | --- | --- | --- | --- | --- | --- | --- | --- | --- | --- |
| XL |  | 2.239 | 3.225 | 3.370 | 5.634 | 2.666 | 1.409 | 4.006 | 1.292 | 2.526 | 3.210 | 1.436 | 2.986 | 3.807 | 3.257 | 1.048 |
| BZ | 0.100^*^ |  | 1.804 | 6.866 | 2.648 | 3.763 | 1.623 | 4.480 | 1.373 | 1.759 | 4.409 | 1.491 | 2.454 | 2.824 | 3.112 | 1.235 |
| LH | 0.072^*^ | 0.122^*^ |  | 4.586 | 3.757 | 3.880 | 3.346 | 6.924 | 2.951 | 8.958 | 4.549 | 3.184 | 11.103 | 14.396 | 5.852 | 1.818 |
| ZW | 0.069^*^ | 0.035^*^ | 0.052^*^ |  | 5.283 | 9.489 | 3.117 | 15.116 | 3.381 | 5.504 | 28.063 | 2.970 | 7.260 | 8.294 | 8.874 | 2.198 |
| CT | 0.042^*^ | 0.086^*^ | 0.062^*^ | 0.045^*^ |  | 4.739 | 2.003 | 5.624 | 2.168 | 2.476 | 4.646 | 2.289 | 3.678 | 7.666 | 3.804 | 1.629 |
| TL | 0.086^*^ | 0.062^*^ | 0.061^*^ | 0.026^*^ | 0.050^*^ |  | 8.685 | 6.362 | 5.323 | 3.092 | 8.801 | 13.979 | 11.839 | 9.942 | 6.645 | 4.573 |
| SY | 0.151^*^ | 0.133^*^ | 0.070^*^ | 0.074^*^ | 0.111^*^ | 0.028^*^ |  | 3.340 | 7.771 | 2.590 | 3.668 | 64.017 | 18.351 | 4.866 | 3.519 | 6.761 |
| LY | 0.059^*^ | 0.053^*^ | 0.035^*^ | 0.016 | 0.043^*^ | 0.038^*^ | 0.070^*^ |  | 3.790 | 5.790 | 15.365 | 4.851 | 10.836 | 11.062 | 15.326 | 3.026 |
| QY | 0.162^*^ | 0.154^*^ | 0.078^*^ | 0.069^*^ | 0.103^*^ | 0.045^*^ | 0.031^*^ | 0.062^*^ |  | 2.639 | 3.156 | 25.286 | 6.677 | 3.178 | 3.578 | 52.493 |
| XB | 0.090^*^ | 0.124^*^ | 0.027 | 0.043 | 0.092^*^ | 0.075^*^ | 0.088^*^ | 0.041^*^ | 0.087^*^ |  | 5.411 | 2.347 | 6.975 | 6.568 | 4.425 | 1.638 |
| HR | 0.072^*^ | 0.054^*^ | 0.052^*^ | 0.009 | 0.051^*^ | 0.028^*^ | 0.064^*^ | 0.016 | 0.073^*^ | 0.044^*^ |  | 4.110 | 11.632 | 11.052 | 7.412 | 2.401 |
| FS | 0.148^*^ | 0.144^*^ | 0.073^*^ | 0.078^*^ | 0.098^*^ | 0.018 | 0.004 | 0.049^*^ | 0.010 | 0.096^*^ | 0.057^*^ |  | 14.774 | 3.924 | 5.865 | 44.155 |
| DG | 0.077^*^ | 0.092^*^ | 0.022^*^ | 0.033^*^ | 0.064^*^ | 0.021^*^ | 0.013 | 0.023^*^ | 0.036^*^ | 0.035 | 0.021^*^ | 0.017 |  | 31.436 | 9.682 | 4.478 |
| XY | 0.062^*^ | 0.081^*^ | 0.017 | 0.029 | 0.032^*^ | 0.025^*^ | 0.049^*^ | 0.022^*^ | 0.073^*^ | 0.037^*^ | 0.022^*^ | 0.060^*^ | 0.008 |  | 8.202 | 2.348 |
| HC | 0.071^*^ | 0.074^*^ | 0.041^*^ | 0.027 | 0.062^*^ | 0.036^*^ | 0.066^*^ | 0.016 | 0.065^*^ | 0.053 | 0.033^*^ | 0.041^*^ | 0.025^*^ | 0.030^*^ |  | 2.521 |
| ZH | 0.193^*^ | 0.168^*^ | 0.121^*^ | 0.102^*^ | 0.133^*^ | 0.052^*^ | 0.036^*^ | 0.076^*^ | 0.005 | 0.132^*^ | 0.094^*^ | 0.006 | 0.053^*^ | 0.096^*^ | 0.090^*^ |  |

Population codes were given in Table S1. ^*^*P* < 0.05: significance level.
